# Assessing the impact of virtual reality on surgeons’ mental models of complex congenital heart cases

**DOI:** 10.1007/s11548-025-03542-7

**Published:** 2025-11-05

**Authors:** Eliot Bethke, Matthew T. Bramlet, Bradley P. Sutton, James L. Evans, Ainsley Hanner, Ashley Tran, Brendan O’Rourke, Nina Soofi, Jennifer R. Amos

**Affiliations:** 1 University of Illinois at Urbana-Champaign—Bioengineering, 1406 W Green St, Urbana, IL 61801 USA; 2 University of Illinois College of Medicine at Peoria, One Illini Drive, Peoria, IL 61605 USA

**Keywords:** Virtual reality, Congenital heart, Mental model, Pre-surgical planning

## Abstract

**Purpose:**

Virtual reality (VR) has attracted attention in healthcare for many promising applications including pre-surgical planning. Currently, there exists a critical gap in comprehension of the impact of VR on physicians’ thinking. Self-reported data from surveys and metrics based on confidence and task completion may not yield sufficiently detailed understanding of the complex decision making and cognitive load experienced by surgeons during VR-based pre-surgical planning.

**Methods:**

Our research aims to address the gap in understanding the impact of VR on physicians’ mental models through a novel methodology of self-directed think-aloud protocols, offering deeper perspectives into physicians’ thought processes within the virtual 3D environment. We performed qualitative analysis of recorded verbalizations and actions in VR in addition to quantitative measures from the NASA task load index (NASA-TLX). Analysis was conducted to identify thematic sequences in VR which influenced clinical decision making when reviewing patient anatomy.

**Results:**

We find a significant increase in reported physician confidence in understanding of the patient anatomy from before VR to after (*p* = 0.012) and identified several common patterns of 3D exploration of the anatomy in VR. Physicians also reported low cognitive stress on the NASA-TLX.

**Conclusion:**

Our findings indicate VR has value beyond simulating surgery, helping physicians to confirm findings from conventional medical imaging, visualize approaches with detail, and help make complex decisions while mentally preparing for surgery. These findings provide evidence that VR and related 3D visualization are helpful for pre-surgical planning of complex cases.

**Supplementary Information:**

The online version contains supplementary material available at 10.1007/s11548-025-03542-7.

## Introduction

Preparing for surgery is a complex task where physicians must synthesize a plan from their review of medical imaging, the patient history, considerations for timing and risk, pragmatic considerations for space and access, and more. During a standard pre-surgical conference, surgeons review patient information and medical imaging which are often presented on 2D displays. As Wu et al. detailed in a 2012 study, the surgeon must then perform the complex task of imagining an approach to take in 3D based on 2D image slices and make important decisions about which tools and techniques they might employ [1]. Certain conditions, including congenital heart diseases, represent an inherently 3-dimensional problem, where the physical structure of the patient’s anatomy is impacted by a defect that impedes healthy function. To address these conditions, congenital heart proceduralists plan out a series of modifications to anatomical structures to produce palliative or corrective outcomes, coordinate with other members of the surgical team, and ultimately form a mental model of the case [2, 3]. The fidelity of the mental model of any patient’s anatomy may be different from physician to physician based on several factors including prior experience, quality of medical imaging, and personal biases [4, 5]. We define a “mental model” to be an internal visual representation of a task or system and “thought processes” as the series of decisions and considerations made in order to build and refine a mental model.

Throughout the pre-surgical planning process, a host of tools are available that seek to make synthesizing a plan easier. These tools include high-resolution displays, human–computer interfaces, and analytical software designed to enhance the interpretation and predictions derived from images and other patient data [6, 7]. One tool that has garnered attention for its potential applications in pre-surgical planning is virtual reality (VR) [3, 8, 9].

Previous studies have evaluated VR tools for surgical applications by employing a combination of 1) task-specific metrics measured within the VR session (e.g., time to completion, accuracy, percent completion, etc.), 2) self-reported metrics via questionnaires, and 3) semistructured or unstructured interviews [8–11]. Many means of studying the impact of VR focus on the general usability and immersion of the tool apart from the impact it is having on the user’s mental models, and others have reported difficulty with applying heuristics to evaluate the usability of VR [12, 13]. For example, Napa et al. recruited physicians to evaluate two VR applications with a think-aloud protocol and NASA-TLX in a pre-designed scenario [8]. However, the physicians are reflecting on hypothetical use-cases and opinions and not reacting to real patient cases, limiting the understanding of the surgical planning process. Reinschluessel et al. investigated VR planning of real patient cases and performed qualitative review of recordings, but their findings focus more on VR as a tool than the surgical decision making process or the progression of the surgeons’ mental models [11].

It can be challenging or impossible to study a user’s mental model from data such as questionnaires, metrics such as time-on-task completion, or by analyzing motion data [4]. Additionally, as Lan et al. report in a 2023 review of medical VR literature, concrete evidence for patient-centric outcomes from VR surgical preparation is sparse, and there are many potential confounding factors that make causal evidence for patient outcomes based on pre-surgical planning difficult to derive from available data [14]. We chose to focus on evaluating how the surgical practitioner’s thought processes in VR affects their mental model of real patient cases to elucidate the ways in which mental models are affected by VR. We developed a methodological approach to elicit detailed descriptions of how users perceive visual information in VR and how that experience impacts their thought processes and clinical expectations.

In this work, we employed a self-paced, self-directed think-aloud format where surgeons could opt to review patient anatomy in VR. Each VR session was administered after traditional imaging had been reviewed, and participants were encouraged to talk through their thought processes as they went. Because these VR sessions were optional, the cases that were captured tended to be more challenging cases which merited the extra time and attention. In order to provide an established baseline from which to build our analysis, we adapted the NASA task load index questionnaire (NASA-TLX) to serve as a pre- and post-VR session interview to quantify perceived effort in VR [15]. The themes and relationships that emerged from the analysis of these VR sessions could translate into many potential benefits, including a deeper understanding of surgical decision making and identification of opportunities for improvements to the tools and technologies used to prepare for surgeries.

This study aimed to assess the impact of VR on physicians’ mental models by analyzing their perceptions of real patient cases and to describe the strategies they employed when navigating complex surgical cases in VR.

## Materials and methods

Three physicians from a large, midwestern hospital system were recruited through email solicitation and snowball recruitment to participate in this effort. The participating physicians had 2, 5, and 27 years of experience operating at the time of the study. Patient and physician consent and enrollment proceeded according to an approved IRB protocol. Patients who had received imaging studies done as part of the standard of care could have their anatomy converted to 3D models in VR at the request of any member of the surgical team as an additional part of their pre-surgical planning after they had reviewed the conventional 2D imaging such as CT or MRI. Upon receiving such a request, support staff de-identified the data and DICOM images were segmented into the organ(s) of interest to create patient-specific 3D model(s). Segmentation focused on creating exact replicas of the anatomy utilizing an FDA approved segmentation software; Materialise (Mimics, Leuven, Belgium) utilizing a process previously described [16, 17]. The 3D model was then loaded into a VR application, Enduvo 1.0 [18]. This process of preparing the models is typically accomplished within 24 h of receiving the request for 3D model. The Enduvo environment allows for insertion of 2D and 3D content and provides basic headset and controller movement and 3D manipulation tools including 3D model scaling, translation, a slicing plane, markers/pointers, and layer transparency adjustments.

When the models had been loaded into the VR environment, the requesting physician was invited to visit a space which has been dedicated to VR and visualization to review the case in VR. The key attributes of a VR system were a GPU-powered workstation with a stereoscopic head-mounted display targeting a machine agnostic environment. There were multiple stations available for physicians to participate, and the hardware used included an HTC Vive 2.0, Oculus Quest 2, and Quest 3 running on an Intel i7-6700 k Windows 10 system with 32 GB ram and an NVIDIA GeForce GTX 1080. MB coordinated and administered the pre-interviews, think-aloud VR sessions, and post-interviews to collect the thoughts of the physicians. The pre-interview involved questions about the physician’s confidence in their understanding of the patient case and their confidence in their existing surgical plan. The physician was then instructed to enter the VR environment to explore the 3D model. As they explored, the physician was prompted to verbalize what they were seeing and what they were thinking about. Unlike a more traditional think-aloud protocol, there were no predefined tasks to be completed, and the VR session was entirely self-directed. When the physician determined they were finished, they exited the VR environment, and post-VR anatomy and plan confidence questions were posed followed by the NASA-TLX questions about perceived mental demand, physical demand, time pressure, overall effort required, and frustration.

**Fig. 1:**
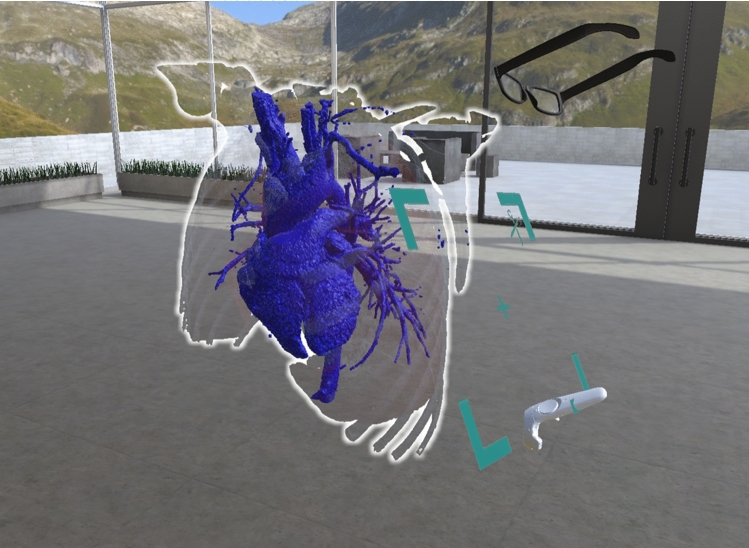
A first-person view of a congenital heart blood pool being reviewed in Enduvo. Participating physicians were recorded talking through their thought processes in this perspective as they explored in 3D

The audio and video recordings of the first-person perspective in VR (see Fig. 1) were subsequently uploaded to a secure, encrypted shared drive where the coders received and analyzed the recordings. A total of 10 sessions were recorded and analyzed, with physician A contributing 6 cases, physician B contributing 2 cases, and physician C contributing 2 cases. Case submissions came sporadically at a rate of around 2–3 per month. The patient cases involved complications including combinations of pulmonary atresia (PA), ventricular septal defects (VSD), major aorto-pulmonary artery collaterals (MAPCAS), patent ductus arteriosus (PDA), coarctation, aortic stenosis, and hypoplasia. For context, [Hospital] has 144 pediatric beds.

**Fig. 2:**
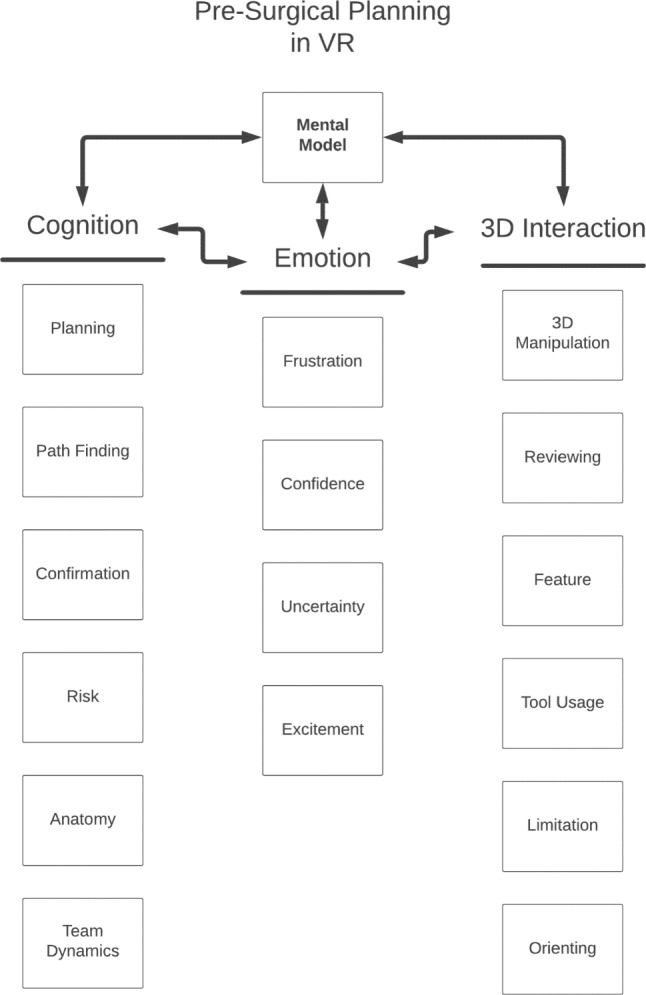
The final coding framework developed to capture significant behaviors in VR sessions. Each box represents a single code. Codes were grouped into three categories (Cognition, Emotion, and 3D Interaction), shown in the columns. Each category and its codes were considered as contributing to constructing and refining mental models through various strategies and thought processes

We employed a qualitative, grounded theory coding methodology to analyze the audio and video recordings of VR sessions [19]. For quantitative analyses, the measurement device and the person analyzing the data are separate, whereas for qualitative methods the two are the same [20]. We opted for a grounded theory approach to avoid the limitations of preconceived frameworks from the literature which predominantly focus on skills measurement or task completion which we felt would be challenging to adapt to the context of mental model formation. Two researchers (EB and JA) screened the first few session recordings to develop an initial set of codes that emerged from the recordings using open coding, which contributed to the development of an initial coding framework. Each code was identified using a short, descriptive name along with a definition of what types of observations would warrant that code, and a unique number by which that code could be encoded. During coding, the physician’s actions in VR (moving, changing tools, manipulating the model) did not necessarily correspond to what they were verbalizing, so we felt it was important to capture both aspects in the video as well as the audio of the recordings. All the pre-session interviews, the VR sessions, and the post-session interviews were then coded using the same coding framework by five independent coders (EB, AH, AT, BO, and NS) who would mark timestamps where codes were identified in each session recording. Codes were iteratively refined at meetings including all coders, with challenges primarily relating to the sporadic case load and the variety of prior experience in coding from each member of the team. During the refinement of codes, we organized the codes into three categories based on what behaviors the codes represented. The physician could be interacting in various ways with the model in VR (3D Interaction), while expressing and emoting verbally (Emotion), or discussing their thoughts on the anatomy or the surgical plan (Cognition). The final set of codes developed can be seen in Fig. 2, and the complete codebook with definitions and examples can be found in Supplementary A.

The decision on how fine-grained to be when coding is typically made by the researcher, but it is often derived from audio transcripts where a complete sentence or thought comprises the “unit of analysis” [19, 20]. Due to the nature of our VR sessions and our interest in capturing not only the verbal accounts of the surgeons but also the ways in which they interact with the 3D environment, we chose not to define a consistent unit of analysis from the audio transcripts alone. Instead, each coder defined their own dynamic unit of analysis where codes were allowed to overlap one another, and the start and end time of each code was rounded to the nearest 5 s from the timestamps of the recordings.

Inter-rater reliability was verified on the codes using Fleiss’ kappa. Fleiss’ kappa accounts for the possibility of agreement between multiple raters occurring by chance and thus provides a more robust metric than simple percent agreement [21]. A kappa of 1.0 implies perfect agreement between coders, a kappa of 0.0 implies agreement occurred at the same expected rate as random chance, and a kappa < 0 implies systematic disagreement. We chose to apply a common scale for interpreting kappa, where < 0 to 0.2 implies very poor agreement, 0.2 to 0.4 implies fair agreement, 0.4 to 0.6 implies moderate agreement, 0.6 to 0.8 implies substantial agreement, and 0.8 to 1.0 implies almost perfect agreement [22].

To reconcile the possibility of coders disagreeing about the timeframes of observations of codes, Fleiss’ kappa was calculated to assess the agreement between coders with each coder serving as reference, thus providing a distinct unit of analysis over which to compute the metric. An agreement was recorded if another coder identified the same code within a 10 s window of the reference coder. Where a coder identified different code(s) at the same window of time as the reference, the difference was recorded for that frame. If another coder had not identified any codes within the window of time identified by the reference coder, a code of ‘0’ was inserted. Coding was captured in a custom spreadsheet (Microsoft Excel), and analysis was performed with custom scripts written in Python. The spreadsheet template and Python scripts are available upon request. Codes were reviewed iteratively until consensus was reached for each session.

In order to evaluate how different strategies and thought processes evolved throughout the VR sessions, transitions in time from one code to another code were extracted from each coder’s codebook. A code transition was defined as any instance where one code began at least 5 s after the previous code had begun. Codes which began at the same were not considered as transitions, but each was considered to transition to the subsequent code(s).

## Results

**Fig. 3:**
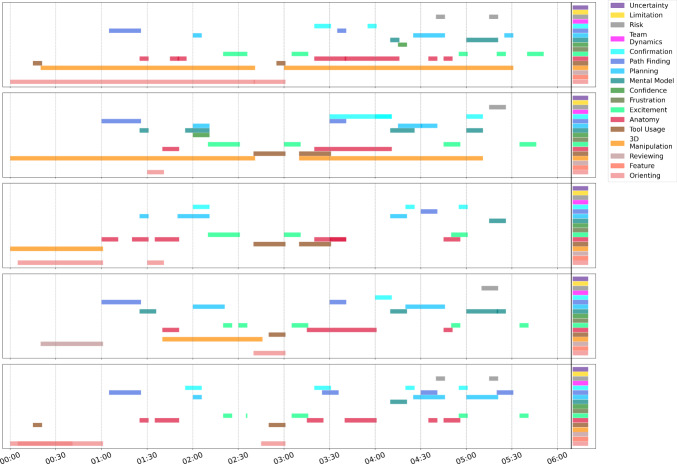
Visualization of the coding of a VR session. Each subplot represents one coder’s codes identified in time (X axis, min:sec), with each colored bar corresponding to a code identified at that time (see legend at right). Not all codes were identified for each session; all codes are shown in legend. This session had a mean Fleiss’ kappa of 0.75

The 10 sessions included for analysis totaled around 2 h and 30 min of time in VR, with a median duration of around 13 min. The shortest VR session lasted approximately 6 min, and the longest session ran for approximately 45 min. A summary of the results from our extended NASA-TLX pre- and post-questionnaires is provided in Tables 1 and 2, reported as the median response and interquartile range (IQR). An example of the coding data from one session is depicted in Fig. 3. The range of agreement for each case depended on which coder was serving as the reference and is shown in Table 3. We considered the prevalence of each code transition, and a summary table of transitions of interest can be seen in Table 4. A full table of all code transitions for all 10 cases can be found in Supplementary B.

**Table 1:** Results from the NASA-TLX questions post-VR experience reported as median (IQR)

NASA-TLX (/10)	Median	IQR
Mental demand	3.00	0.75
Physical demand	2.50	1.00
Time demand	1.00	0.75
Performance	10.0	0.75
Effort	3.00	2.50
Frustration	1.00	0.00

Questionnaire was implemented with Mental, Physical, and Time demand rated as 1 = very low effort/pressure and 10 = very high effort/pressure. Performance was rated from 1 = failure to 10 = perfectly satisfied, Effort was rated from 1 = very low total effort to 10 = very high total effort, and Frustration was rated from 1 = very low frustration to 10 = very high

**Table 2:** Pre- and post-VR confidence out of 5, reported as median (IQR) for all 10 sessions

	Pre-VR	Post-VR	p (Welch’s t test)
Confidence in Anatomy (/5)	**4 (0.75)**	**5 (0.75)**	0.012*
Confidence in Approach (/5)	4 (0.75)	4 (1.00)	0.310

The bold values indicates statistical significance. *p < 0.05, **p < 0.01, ***p < 0.001

**Table 3:** Fleiss’ kappa score ranges (min and max) for all 10 sessions across 5 raters

Session	Min κ	Max κ
1	0.621	0.727
2	0.479	0.572
3	0.471	0.673
4	0.547	0.693
5	0.695	0.782
6	0.502	0.681
7	0.478	0.662
8	0.709	0.819
9	0.582	0.655
10	0.661	0.682

0.2—0.4 was considered fair agreement, 0.4—0.6 was considered moderate agreement, and 0.6—0.8 was considered good agreement

**Table 4:** Most prevalent code transitions from all cases

Code transition	Shared count
3D Manipulation → Anatomy	199
Tool Usage → D Manipulation	194
Orienting → Tool Usage	185
Orienting → Anatomy	176
Anatomy → Confirmation	154
Orienting → 3D Manipulation	131
3D Manipulation → Orienting	117
Anatomy → Planning	117
Tool Usage → Anatomy	115
Anatomy → Orienting	98

Transitions were identified for each coder, and the intersection of the sets of transitions from each pair of coders was summed to give these totals across all 10 sessions

Four major themes emerged from our analysis which provide insight into the strategies employed by each physician as they prepared for their complex congenital heart surgical cases. These themes were identified both from the codes which emerged, as well as through notes captured by each coder being cross-compared. The behaviors and verbalizations, which provided both comparative and contrasting reflection, produced the following themes: 1) decision making arising from sequences of exploration, 2) 3D exploration helping to confirm and refine understanding of anatomy, 3) emotion as an indicator for opportunities, and 4) a physician’s confidence masks more complex thought processes.

### Theme 1: decision making arises from sequences of thought and exploration

Throughout the refinement of the coding scheme and the analysis, we began to focus not only on the frequency of codes, but how the codes interacted with one another over time. When examining the patterns of code transitions in all sessions, we noted that transitions in time from one code to another appeared with different prevalence and were not always symmetric. For example, statements made about verifying anatomical structures (Confirmation) which led to statements about perceptions of the patient case (Mental Model) were identified 36 times in 10 cases, whereas the reverse (Mental Model leading to Confirmation) was only found 17 times in the same 10 cases, roughly half as frequently. We might presume then that the physicians have a goal of exploring the 3D anatomy to confirm or align their mental image of the patient anatomy which they had reviewed in 2D, which then led to them discussing their thoughts about the case, resulting in the one-sidedness. Table 4 shows the most prevalent code transitions across all sessions for further context.

### Theme 2: 3D exploration helps confirm and refine understanding of anatomy

Throughout the 10 sessions, our codes that indicate 3D interaction (motion, manipulation of the 3D model, and 3D tool usage) were often co-located with a verbal discussion of the patient anatomy (Anatomy) as well as the surgical plan (Planning). As seen in Table 4, codes involving 3D movement and manipulation were the most prevalent codes identified. Manipulation of the model by slicing, scaling, or rotation (3D Manipulation) most commonly preceded discussions of patient anatomy (Anatomy), at an average identification rate of nearly 20 instances per case. Early on, we identified an interesting behavior where physicians would verbally indicate their intention to trace a path from one anatomical feature to another (Path Finding), which is another feature of the 3D environment that makes this type of navigation more intuitive than in a 2D stack mode view. We provide several quotes from physicians as they enacted path finding and generally explored the anatomy:

*“Nothing like walking through the band and looking down the barrel of both PAs.”*

*“I can walk down the course of [the pulmonary artery], right down to the lower lung.”*

*“I immediately have a greater appreciation. It had been described as having an inlet extension, but it’s really sitting well below the tricuspid.”*

Another similar observation was in how the surgeons moved between exploring the anatomy and talking through their surgical plan. Discussion of the surgical plan (Planning) was frequently followed by enthusiastic expressions (Excitement, 30 times) and statements about verifying anatomical structures (Confirmation, 26 times). Physicians would often trace a path through the anatomy (Path Finding) after discussing the patient anatomy more generally (Anatomy, 69 times) but would also spontaneously chart paths as they explored the 3D model (3D Manipulation, 41 times). A different common pattern emerged as physicians dealt with uncertainty (Uncertainty), which most commonly presented after discussing the patient anatomy (Anatomy, 80 times) and led to further movement and exploration of the VR environment (3D Manipulation 30 times, Orienting 28 times).

These sequences highlight how the surgeons are approaching the VR tool to enhance their surgical planning, looking to explore and confirm the patient anatomy in 3D and refine their mental model through exploration. We have extracted all the most common code transitions involving the Mental Model code in Table 5. Of all the codes, 3D Manipulation, Anatomy, Tool Usage, Orienting, and Confirmation appear most frequently, which may seem obvious given the nature of the exercise being an exploration of patient anatomy in 3D. However, specific patterns of how and when surgeons chose to discuss their surgical plan and their mental model of the case, along with how they expressed emotion throughout the VR session, show evidence of purposeful sequencing derived from goal-oriented exploration. The sequences point to dynamic strategies of 3D exploration leading to confirmation, and refinement of uncertainties with continued exploration and path finding helping to methodically construct detailed mental models of the patient anatomy.

**Table 5:** Code transitions calculated as described in Table 3, but only for transitions involving the code, “Mental Model”

Code transition	Shared count
Mental Model → Anatomy	**61**
3D Manipulation → Mental Model	**47**
Anatomy → Mental Model	**46**
Confirmation → Mental Model	**36**
Mental Model → Orienting	**20**
Mental Model → Confirmation	**17**
Mental Model → 3D Manipulation	**16**
Path Finding → Mental Model	**14**
Planning → Mental Model	**13**
Uncertainty → Mental Model	**11**
Mental Model → Path Finding	**10**
Mental Model → Excitement	**10**

The bold values indicates statistical significance

### Theme 3: emotion as an indicator for opportunities for VR

Participants frequently became excited (Excitement) when exploring the anatomy (Anatomy, 105 times), making statements such as, *“That’s the one I want, right there!”*, and *“See, this image is great!”*. By contrast, exclamations of frustration (Frustration) were most associated with the VR tool itself (Tool Usage 48 times, 3D Manipulation 29 times, and Orienting 26 times), and much less frequently with the nature of the case (Anatomy 10 times). We take some measure of caution interpreting these results, as our participants have volunteered to review cases in VR, introducing possible selection bias where physicians who were more likely to opt to review a case in VR would be more inclined to be excited and positive about the experience. A quote highlighting one surgeon’s reflection on their VR experience toward the end of a session (below) highlights how excitement cued the research team into key moments where the various strategies of exploration, path finding, and confirmation of 2D imaging often led to an improved mental model of the anatomy.

*“I wanted to define exactly where I was going to bring the descending aorta up to, on the ascending and arch segment to avoid any residual narrowing or obstruction and this shows it quite nicely! I could almost draw a line right where I’m going to plug into the descending segment!”*

### Theme 4: confidence may mask more complex decision making during planning

When reviewing our pre- and post-session survey items, we found that in 6 out of 10 sessions, confidence in surgical approach increased, typically from 4/5 to 5/5 (3/10 sessions) or 3/5 to 4/5 (2/10 sessions). For the remaining 4 sessions, the physician’s self-reported confidence in their initial surgical approach stayed the same (2/10 sessions) or decreased (2/10 sessions) after reviewing the anatomy in VR. The reported confidence in understanding of the patient anatomy had just 2 sessions where it stayed the same (1/10 sessions) or decreased (1/10 sessions). A Welch’s t test showed no statistically significant change in reported confidence in the surgical approach from before to after the VR session (p = 0.310). The same test of reported confidence in understanding of the anatomy from before and after VR does show a significant increase after VR (p = 0.012).

## Discussion

From the NASA-TLX pre- and post-session results, we saw high accomplishment of goals (median score 10/10), low mental demand (median score 3/10), low physical demand (median score 2.5/10) and low time pressure (median score 1/10) all with low variability, indicating our physicians perceived the VR sessions to be easy to navigate and complete. In the latter 7 sessions, the physicians were asked to report their mental demand of reviewing the anatomy in surgical conference (traditional 2D view), which resulted in a median reported score of 6/10 and an IQR of 1.5. A Welch’s t test comparing the mean mental demands in surgical conference vs VR for the 7 sessions where this was collected shows a significant decrease (p = 0.016), indicating the physicians had an easier time understanding the patient anatomy in VR than by reviewing traditional medical imaging. From the analysis of the VR sessions, we found viewing the data from the perspective of sequences (Theme 1) to be particularly useful in evaluating our other major themes.

### Exploration in 3D

In our study, the main focus for these physicians appears to be centered on developing a richer understanding of the patient anatomy, which is a finding in line with other studies [8, 9, 11, 23]. When we looked at how the surgeons used the VR tool, we saw a mix of different strategies to explore the anatomy to build that understanding. Most prominently, the surgeons constantly slice, translate, rotate, and scale the 3D model as they move around. We observed each participant tracing paths through the model, and taking multiple approaches to reach the defect or structure of interest with high spatial dynamism. As they talked aloud, they would mention how they were updating their understanding of the case, where they were uncertain, and how they planned to proceed. Finally, we take this all in the context that the surgeons are volunteering to review these cases in VR, with no structured tasks or objectives guiding their experience in VR other than their own reflection on the patient case. We believe this adds additional significance to the themes we have identified in the common behaviors we see in VR from our qualitative analysis. In future work, we intend to further explore potential relationships between surgeon experience, the type of surgery (e.g., oncology, spinal, brain, thoracic, adult, pediatric, etc.), and other potential factors that may influence the purpose and role VR serves as a pre-surgical planning tool.

### Presence of emotion

A prominent aspect of our coding scheme that emerged from our data was the prevalence of emotions, which distinguishes this effort from many contemporary studies. As we presented in our results, we found that most positive emotions (Excitement, Confidence) were associated most often with concurrent exploration of the anatomy and planning for the surgery (Anatomy, Planning), while the more negative emotions (Frustration) were most commonly associated with the VR tool itself. Again, this aligns with prior work which has found there to be some learning curves with VR tools and interfaces [24]. We also note the common trend we found where 8 out of 10 sessions ended with an Excitement code being identified. So, while there are opportunities to enhance VR tools to be easier to use, more intuitive, and more responsive, we found the prevalence of positive emotion to indicate that even non-optimal VR environments may provide some emotional benefit to the physician for pre-surgical planning. We are especially interested in exploring the role of physician experience in how emotions drive certain perceptions of cases, as we noticed our most experienced surgeons tended to be distinctly expressive and positive about their interactions in VR. With this small sample size, we cannot make specific claims, but we are interested in expanding our participant pool in future to allow for statistical models to be developed and test this observation more broadly.

### Confidence as a mask

We were interested in the result that while physicians’ confidence in their understanding of the patient anatomy increased significantly (p = 0.012), their reported confidence in the surgical approach did not significantly change (p = 0.310). An F test showed a significant decrease in variability in confidence in the anatomy from pre- to post-VR as well (p = 0.026), with all sessions reporting a 4/5 or 5/5 after VR. This was especially interesting given prior work which has found that surgical approaches being considered narrowed and confidence increased after surgeons reviewed cases in VR [25, 26]. In 4/10 sessions we conducted, the confidence in the surgical approach stayed the same or decreased. In the 2 sessions where confidence in the surgical approach stayed the same, the confidence was relatively high (4/5 or 5/5). We interpret this along with the observed behaviors in VR that physicians are treating the VR tool to integrate more perspective on the anatomy of the case and are not necessarily using the tool to rehearse the surgery itself. The abundance of time participants chose to spend in VR on freely exploring the anatomy and confirming their existing conceptions of the case may help explain why confidence or task-specific metrics may be poor measures for understanding the impact of VR for pre-surgical planning, especially given the low mental, physical, and time demand reported in the NASA-TLX.

It is common to find statements focusing potential use-cases of VR to education and training, where trainees may serve as a clearer target for practice and exploration [9, 14, 27]. We believe some of this may stem from the field of surgery selecting confident, motivated individuals, thus narrowing the utility of questions around confidence. However, when we dug a bit deeper into the pre- and post-questionnaire, a more complex narrative about surgeon confidence began to unfold. For example, physician A responded to our pre-session questionnaire item, “On a scale of 1–5, how confident are you in your surgical approach for this case?” as follows:

*“I’m confident that I can do this. The real question is, can I close one hole and be done and this patient will be fine? Or do I need to go after these others? And if so, how am I going to find my surgical confidence?”*

This quote highlights how a physician can identify a need or question (e.g., to decide which defects need to be addressed), which establishes a goal for their VR session. The surgeon’s education and training, prior experience, and the skillset all give them the confidence to synthesize their options, weigh complex decisions, and form strategies as they explore the anatomy in 3D space. These VR sessions provide perspective and time to evaluate and form strategies in a way that is not strictly rehearsal as the goal for some other VR interventions [28–31]. Rather, the free exploration serves to enhance the mental model of how their approach might change given the patient’s anatomical presentation. In the post-session interview from the same session as the quote as above, the surgeon was asked why they reported an increase in confidence in the anatomy if not the surgery, and we hear this process explained plainly:

*“Well, I was able to visualize at least that mid-septal area a little better than I could see it on the Echo. So the large VSD [Ventricular Septal Defect], I had a pretty good working image of that in my head already, but the others I did not. The fact that the ones even lower down in the septum, even in VR are not clear in a sense [that if] there’s not a straight hole right there, I would argue that they’re not going to be that significant and probably don’t need to be addressed surgically.”*

Later, when the same surgeon was probed about why they felt the VR session had increased their confidence, they remarked:

*“It’s a better understanding of the anatomy, it’s just a more graphic representation in my mind of what I’m going to find tomorrow. And I’m confident that it’s going to be exactly that.”*

### Limitations

We believe our self-directed think-aloud protocol and qualitative analysis presents an advancement in how VR applications can be evaluated for surgical planning. Our study presents several limitations as well, which we acknowledge here. Our method of recruitment for this study was entirely voluntary, and thus, surgeons opting to submit cases may have already perceived the benefit of VR as a pre-surgical planning tool and may have been more likely to express positive emotion about the experience. In that regard, we have attempted to avoid making any claims of causal relationships to specific outcomes and focus instead on reporting the common behaviors and strategies we observed. As a result of the complexity of the cases, our inter-rater reliability metric scores (Fleiss’ kappa) were comparatively low for some cases. This was additionally challenging with 5 coders working to find consensus for how codes should be applied, requiring a significant time investment of approximately 4 months of review.

Additionally, in one session where author MB was unavailable to administer the VR session, and we found very little variety in the codes, with very few verbal expressions present for that case. This potentially highlights how our method may be sensitive to the expertise and rapport between the participant and the administrator of the session. We also acknowledge that the number of surgeons participating in this study was quite small (n = 3). However, each surgeon contributed at least 2 cases, and we feel the total duration of interactions in VR still provides a significant dataset to work from to establish common trends and behaviors. Finally, this entire effort would not have been possible without a host of tools and technologies enabling the 3D model creation, the space and hardware to host these VR sessions, and the technicians to operationalize the software and hardware which may represent a significant barrier to adopting these methods more broadly.

## Conclusion

This study represents a new approach to identifying and quantifying surgeons’ strategies for how they use the 3D information in VR to enhance their mental model of patient anatomy during pre-surgical planning. Our evaluation approach offers numerous advantages compared to traditional task-based metrics or statistical assessment of survey data. By employing an unguided structure of observation anchored to the pre- and post-NASA-TLX questionnaires, we demonstrate how patterns of behaviors emerge in VR, and not only reinforce preconceived notions of pre-surgical planning but also shed light on the specific strategies and approaches surgeons employ in VR to enhance their understanding of the patient anatomy. The cases volunteered were complex and prompted our physicians to explore dynamically and adjust or refine their mental model of the case. The strategies we observed in VR were often inherently three-dimensional, which led to Path Finding, Confirmation of structures identified in 2D, and exploring the model thoroughly with Tool Usage and 3D Manipulation of the model. In future, we hope to expand this type of analysis to more physicians and more fields of surgery to continue to identify opportunities for technologies like VR to make a positive impact on surgeon preparedness for complex surgical cases.

## Supplementary Information

Below is the link to the electronic supplementary material.

Supplementary file1 (XLSX 13 KB)

Supplementary file2 (XLSX 14 KB)
